# Comparison of hook plates *vs*. locking plates for Neer type IIB fractures of lateral end clavicle: A systematic review

**DOI:** 10.1016/j.cjtee.2024.03.012

**Published:** 2025-02-19

**Authors:** Ravi Patel, Muhammad Murtaza Khan, William Gibson, Robin Banerjee, Asif Pardiwala

**Affiliations:** a Department of Trauma and Orthopaedics, Robert Jones and Agnes Hunt Orthopaedic Hospital, Oswestry, United Kingdom; b Department of Trauma and Orthopaedics, The Princess Royal Hospital, Apley Castle, Telford, TF1 6TF, United Kingdom; c Department of Trauma and Orthopaedics, Royal Shrewsbury Hospital, Mytton Oak Rd, Shrewsbury, SY3 8XQ, United Kingdom; d Department of Trauma and Orthopaedics, Scunthorpe General Hospital, Scunthorpe, DN15 7BH, United Kingdom

**Keywords:** Complications, Coraco-clavicular ligament, Distal clavicle fracture, Hook plate, Locking plate, Reconstruction, Union

## Abstract

**Purpose:**

Surgical management of the lateral end of clavicle fractures has been a challenge for orthopedic surgeons considering the high rate of non-union. There has been no right and wrong answer to these types of fractures and many methods discussed in the literature, but the 2 most used bony procedures are hook plate and locking plate with or without the use of supplementary soft tissue procedures. The available evidence, in this case, is scarce with questionable reliability. The idea of this systemic review is to promote evidence-based practice when choosing between the 2 implants for this fracture. This study aims to review the results by performing a systemic review of the literature comparing the results of locking plate *vs*. hook plate for the lateral end of clavicle fracture fixation with an emphasis on outcome and associated complications.

**Methods:**

A search of the literature was made with the keyword “clavicle” in PubMed/Ovid Medline/Embase and University of Edinburgh online library “discover Ed”. A total of 4063 articles were identified including case series (with at least 3 cases) and review articles focusing on locking plate alone, comparisons of locking plate and hook plate, or hook plate alone. Articles were excluded if they were not published in English, focused on pediatric studies, or consisted only of book chapters. Studies examining tension band wiring, soft tissue procedures for fracture fixation, arthroscopic-assisted procedures, additional soft tissue procedures along with plate fixation, and fracture dislocation of the lateral end of the clavicle were also excluded. The search was then narrowed down to 21 articles after consideration of inclusion and exclusion criteria. A detailed review of the surgical methodology further excluded additional soft tissue procedures, resulting in a final selection of 15 studies. The quality of the studies was assessed using the Modified Coleman Score by the authors.

**Results:**

A total of 15 studies related to Neer type II fracture met the inclusion criteria. However, 2 other studies also included type V fracture as well. The mean age of patients in these studies was 32 years. The mean follow-up period was 24.3 months (ranging from 6 to 65 months). The time of radiological union was documented from 2 to 4.5 months. Constant and disabilities of arm, shoulder, and hand scores were most used as the criteria for patient outcomes. The size of the lateral fragment that can accommodate/provide bicortical fracture was documented in only 3 studies. The mean incidence of removal of hook plate was 86.9%. In contrast, the mean incidence of removal of locking plate was 27.0%. Superficial wound infection was documented in 5 studies and deep wound infection was seen in 1 study. The mean union rate for hook plate was 97.0% compared to 100% for locking plate. Complications associated with hook plate have been documented in 11 studies. The most commonly reported incidence of complication was acromial osteolysis. The quality of studies was assessed using modified Coleman score. Other than 2 studies that were considered for the study that met the “fair” standard all of them were considered “poor” based on the modified Coleman score.

**Conclusion:**

Both hook plate and locking plate provide acceptable operative treatment options for the lateral end of clavicle fracture. However, a consideration of surgeons’ experience, the likelihood of a second operation, and the size of the lateral fragment should be considered when choosing between the 2 types of implants.

## 1 Introduction

The common etiology of lateral end of clavicle fracture is direct trauma to the shoulder. Neer classification is commonly considered for the lateral end of clavicle fracture. Neer classified these fractures into 3 types with type II being further divided into 2 types A and B based on integrity or disruption of the coracoclavicular ligament. Type II fractures have a high rate of non-union and are considered unstable and prone to non-union with conservative treatment. Neer classification system is widely accepted as it helps in differentiating unstable from stable fracture patterns.^1^

The forces acting on the fracture fragment cause displacement of fracture fragments. The proximal fragment is displaced inferiorly due to the weight of the upper extremity.^2^ The lateral end of clavicle fracture accounts for 25% of all clavicle fractures. The management of Neer type II fractures of the distal clavicle is controversial, with no clear consensus as to whether non-surgical or surgical management results in better long-term outcomes.^3^ Considering the small size of the lateral fragment, the ideal fixation option is debatable. Non-union rate was documented as 22%–50% with 14% developing symptomatic non-union when opting for conservative treatment. Various surgical treatment options have been discussed in the literature which involve coracoclavicular screws, coracoclavicular ligament reconstruction, Kirschner wires, tension band wiring, hook plate, and non-locking and locking plate options.^4^^,^^5^

Considering the challenge associated with the fixation of these fractures and the background mentioned above, a comprehensive systemic review of the literature was performed to assess the quality of available evidence based on the use of these 2 implants. The evidence regarding the use of fixation methods has been poor. There are quite a few level IV studies published in the literature. To help surgeons make the right choices and improve the available evidence, we conducted a systematic review of the literature on the use of locking plate fixation or hook plate as the only treatment option with no other soft tissue. Moreover, modified Coleman score was used to develop objective evidence to evaluate the quality of studies being published.

According to studies, hook plate has a high rate of union, but hook plate has been reported to cause implant-related complications, such as acromion osteolysis and peri-implant fractures.^5^ Moreover, most patients are likely to undergo a second surgery to avoid any long-term complications associated with the use of hook plate. Therefore, a major drawback or consideration when using this implant is to plan the elective removal of metal work over some time to avoid any long-term complications.

## 2 Methods

A search of the literature was made with the keyword “clavicle” in the databases of PubMed/Ovid Medline/Embase and University of Edinburgh online library-discover Ed. A total of 4063 articles were identified. The search was then narrowed down using the terminology of “distal end of clavicle/lateral end of clavicle”, “hook plate”, and “pre-contoured locking plate fixation for displaced lateral clavicle fracture”.

“Distal end of clavicle facture” revealed 58 papers in total and out of these 8 were found suitable for review. “Lateral end of clavicle fracture” revealed 104 articles and out of these 2 were included in this study. “Pre-contoured locking plate fixation for displaced lateral clavicle fracture” revealed 152 papers and out of these 21 met the inclusion criteria. A detailed review of the surgical techniques used in these studies revealed that soft tissue surgery/additional fixation methods were occasionally used as needed. Based on the criteria agreed upon for selection and after discussion with the co-authors, 15 studies were included in our systemic review.

### 2.1 Inclusion & exclusion criteria

**Fig. 1:**
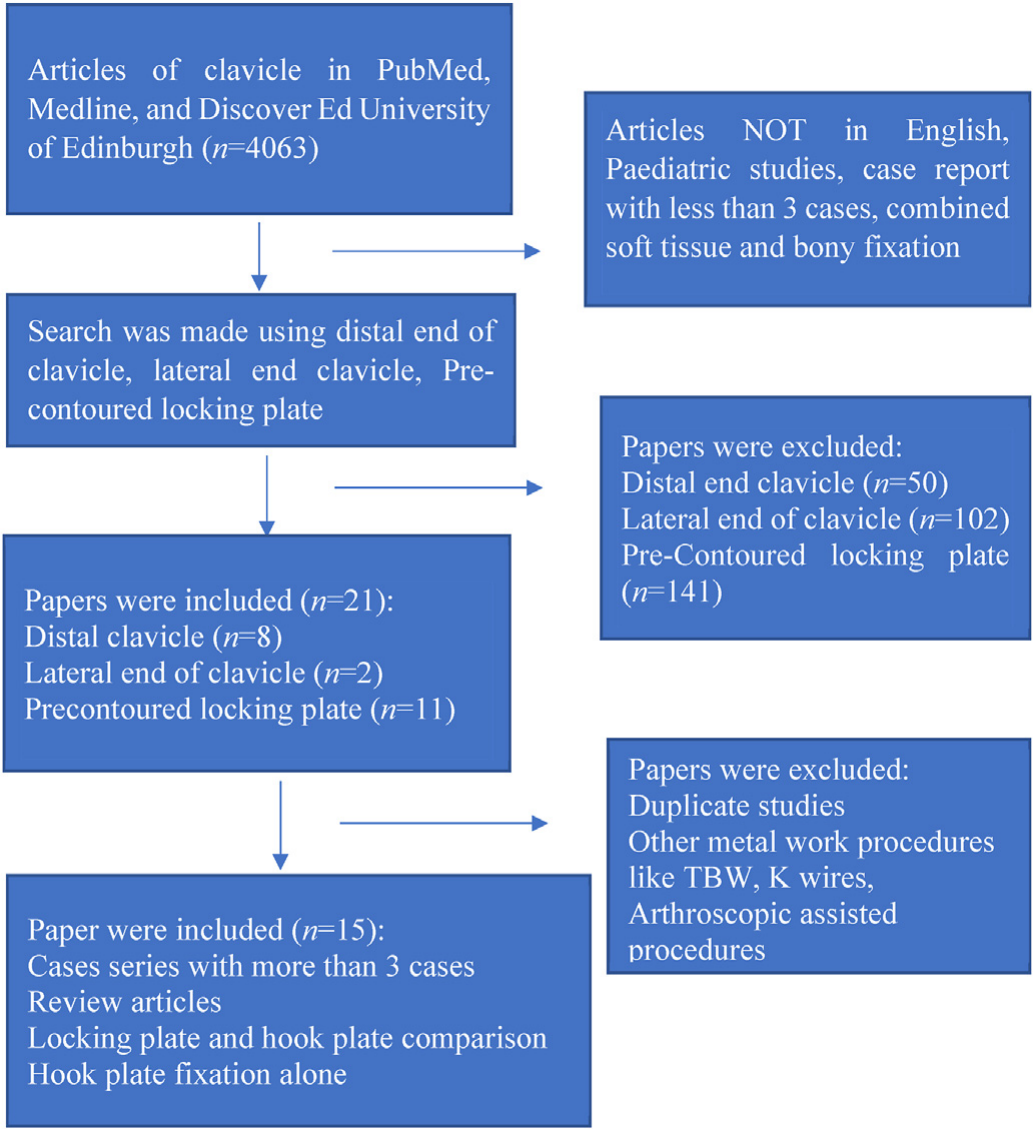
The flowchart of this study. TBW: tension band wiring.

Inclusion and exclusion criteria in this study were discussed with the co-authors and once consensus was made the fate of the article was decided to reduce the risk of selection bias. The inclusion criteria were: case series (at least 3 cases) and review articles related to locking plates alone, locking plate and hook plate comparison, or hook plate alone. Articles not published in English, related to pediatric studies and with only chapters of books were excluded. The study of tension band wiring and soft tissue procedures for fracture fixation, arthroscopic assisted procedure, addition soft tissue procedure along with plate fixation, and fracture dislocation of lateral end of clavicle were excluded. Comparison of hook plate with soft tissue procedure/additional procedure with plating (screws/wires) and tension band wiring/hook plate were not chosen (Fig. 1).

### 2.2 Quality of study

The assessment was based using the Coleman methodology score (Table 1). The studies were then categorized according to their scores, with scores between 85 and 100 being excellent, 70–84 being good, 50–69 being fair, and below 50 being poor. The assessment of the study quality was also made by 2 authors independently on 2 different occasions to avoid bias. When there was a discrepancy in the assessments of the 2 authors that could change the grading status of the study, the opinion of a third author was sought, and the opinion of the third author was considered final.

**Table 1:** Modified Coleman score.

Category	Modified Coleman score
Study size (number of patients)	
<15	0
15-24	4
25-40	7
40	10
Mean follow-up (year)	
<1	0
1-2	4
2-5	7
5	10
Number of different surgical techniques	
Not stated	0
Several techniques but clearly stated	5
1 technique but >90% receiving 1 technique	7
One technique	10
Study type	
Case report	0
Case series	2
Retrospective comparative study	3
Prospective cohort study	10
Randomized control trial	15
Postoperative management/rehabilitation	
Not formalized	0
Yes, but unclear	2
Yes, and clear	5
Description of surgical technique	
Inadequate/not clear	0
Fair (technique only stated)	3
Detailed (description of materials used)	5
Precise and detailed (pictures/diagrams)	10
Complication discussed	
Unclear/not mentioned	0
Yes, but unclear	2
Yes, and clear	5

### 2.3 Outcome measure

The outcome measures for the studies under consideration included several key parameters. The size of the lateral fragment was measured by the length available for bicortical screw fixation. Union was assessed radiologically, focusing on cases showing no signs of union after 6 months, particularly with 2 types of implants. Infection rates were classified into superficial wound infections, affecting only the skin and not requiring implant removal, and deep wound infections, which involved the implant and necessitated its removal. The incidence of impingement was noted following hook plate insertion. Additionally, implant removal was tracked, with a focus on the duration from surgery to removal and its frequency. These criteria were standardized by converting results into months and percentages, providing uniformity and clarity in evaluating the findings across the included studies.

## 3 Results

A total of 15 studies met the inclusion criteria, all of which involved Neer type II fracture. However, 2 other studies also included type V fracture as well. The mean number of patients in these studies was 32 with a range ranging from 12 to 67. Except for 1 randomized study, all were retrospective studies (Table 2).

**Table 2:** Study details.

Authors	Description of surgical implant (*n*)	Subject selection	Number of patients	Type of study
Chen et al.^1^	Hook (12) *vs*. locking plate (19) (mini, small fragment)	Neer type IIB, V	31	Retrospective review
Şükür et al.^2^	Hook plate (16)	Neer type IIB	16	Retrospective review
Kapil-Mani et al.^3^	Precontoured locking plate (46)	Neer type IIB	46	Retrospective review
Vaishya et al.^4^	Precontoured locking plate (32)	Neer type IIB	32	Retrospective review
Tambe et al.^5^	Hook plate fixation (18)	Neer type IIB	18	Retrospective review
Govindasamy et al.^6^	Superior anterior locking plate (12)	Neer type IIB	12	Retrospective review
Erdle et al.^7^	Locking plate (13) *vs*. hook plate (19)	Neer type IIB	32	Retrospective review
Ochen et al.^8^	Hook plate (23) and superior clavicle plate with lateral extension (53)	Neer type IIB, V	67	Retrospective review
Haider et al.^9^	Hook plate (22)	Neer type IIB	20	Retrospective review
Tiren et al.^10^	Hook plate (28)	Neer type IIB	28	Retrospective review
Lopiz et al.^11^	Hook plates (24)	Neer type IIB	24	Retrospective review
Muramatsu et al.^12^	Hook plate (15)	Neer type IIB	15	Retrospective review
Renger et al.^13^	Hook plate (44)	Neer type IIB	44	Retrospective review
Wang et al.^14^	Hook plate (33), locking plate (31)	Neer type IIB	64	Randomization

The mean follow-up was 24.3 months with a range from 6 to 65 months. The time of radiological union was documented from 2 to 4.5 months. However, 2 studies did not specify the time frame of the union. Constant and disabilities of arm, shoulder and hand scores were most used as the determinate of patient outcomes. The size of lateral fragment which can accommodate/provide biocritical fracture was documented in only 3 studies (Table 3).

**Table 3:** Size of lateral fragment.

Authors	Follow-up period (month)	Mean time of union (months)	Functional outcome score	Lateral fragment size (mm)
Chen et al.^1^	36	4	Quick DASH	Not mentioned
Şükür et al.^2^	12	4	UCLA score	Not mentioned
Kapil-Mani et al.^3^	12	Not mentioned	Constant Murley score, UCLA shoulder score	Not mentioned
Vaishya et al.^4^	6	3.5	VAS and DASH score	Not mentioned
Tambe et al.^5^	25	5	Pain score, constant score	Not mentioned
Govindasamy et al.^6^	12	2	DASH and OSS	Not mentioned
Erdle et al.^7^	54	4.2	Constant, OSS, Taft score and subjective shoulder value	19.5 ± 5.6 HP, 23.9 ± 10.1 LP
Ochen et al.^8^	12	4	Quick DASH, NRS activity	15 ± 4 HP, 20 ± 8 LP
Haider et al.^9^	39	4.5	American shoulder elbow surgeon	Not mentioned
Tiren et al.^10^	65	4.5	DASH and Constant score	Not mentioned
Lopiz et al.^11^	12	3.5	UCLA score, Constant score	Not mentioned
Muramatsu et al.^12^	15.5	4	Constant Murley score	Not mentioned
Renger et al.^13^	27.4	Not mentioned	Constant Murley score	Not mentioned
Wang et al.^14^	12	3	VAS, constant Murley score, UCLA	31.17 ± 3.97 HP, 33.21 ± 4.15 LP

DASH: disabilities of arm, shoulder and hand, UCLA: University of California and Los Angeles, VAS: visual analogue scale, OSS: Oxford shoulder score; NRS: numeric rating scale; HP: hook plate, LP: locking plate.

The mean incidence of removal of hook plate was 86.9%. All but 1 study recommended removal of the implant, and the most common reason for implant retention was that patients refused to undergo a second surgery because they were completely asymptomatic. In contrast, the mean incidence of removal of locking plate was documented to be 27%. The most common etiology in the reviewed studies was skin irritation, followed by patient wishes. Superficial wound infection was documented in 5 studies and deep wound infection was seen in 1 study. The mean union rate of hook plate was 97% compared to 100% for locking plate (Table 4).

**Table 4:** Complications.

Authors	Implant removal incidence, %		Superficial wound infection	Deep wound infection	Union rate, %	
	Hook plate	Locking plate			Hook plate	Locking plate
Chen et al.^1^	83	37	Not recorded	Not recorded	100	100
Şükür et al.^2^	62	N/A	3	Not recorded	100	100
Kapil-Mani et al.^3^	N/A	0	1	Not recorded	N/A	100
Vaishya et al.^4^	N/A	0	Not recorded	Not recorded	N/A	100
Tambe et al.^5^	94	N/A	Not recorded	1	89	N/A
Govindasamy et al.^6^	N/A	0	1	Not recorded	100	100
Erdle et al.^7^	100	77	Not recorded	Not recorded	95	100
Ochen et al.^8^	100	42	Not recorded	Not recorded	95	98
Haider et al.^9^	100	N/A	Not recorded	Not recorded	95	N/A
Tiren et al.^10^	96	N/A	1	Not recorded	96	N/A
Lopiz et al.^11^	95	N/A	Not recorded	Not recorded	96	N/A
Muramatsu et al.^12^	80	N/A	Not recorded	Not recorded	100	100
Renger et al.^13^	68	N/A	2	Not recorded	100	N/A
Wang et al.^14^	78	32	Not recorded	Not recorded	100	100

Complications associated with hook plate have been documented in 11 studies with a variable incidence of subacromial impingement, osteolysis, acromioclavicular joint arthrosis, and peri-implant fracture (Table 5). The most common reported incidence was acromial osteolysis.

**Table 5:** Complications secondary to metal work (%).

Authors	Subacromial impingement	Acromial osteolysis	ACJ arthrosis hook plate	Peri-implant fracture hook plate
Chen et al.^1^				
Şükür et al.^2^		42	62	
Tambe et al.^5^		27	27	
Erdle et al.^7^		26.30	42.10	1
Ochen et al.^8^				
Haider et al.^9^				1
Tiren et al.^10^	32	25	14	
Lopiz et al.^11^	4	12.50	37.50	1
Muramatsu et al.^12^				
Renger et al.^13^		6.80	4.50	
Wang et al.^14^			9	

ACJ: acromioclavicular joint.

Study quality was assessed using modified Coleman score by 2 authors on 2 different occasions, which categorized study quality as excellent, good, fair, and poor. All studies were considered “poor” except for 2 studies that met the “fair” criterion (Table 6).

**Table 6:** Quality of studies.

Serial No.	Authors	Modified Coleman methodology	Quality of study assessment
1	Chen et al.^1^	32	Poor
2	Şükür et al.^2^	33	Poor
3	Kapil-Mani et al.^3^	57	Fair
4	Vaishya et al.^4^	43	Poor
5	Tambe et al.^5^	32	Poor
6	Govindasamy et al.^6^	34	Poor
7	Erdle et al.^7^	33	Poor
8	Ochen et al.^8^	45	Poor
9	Haider et al.^9^	33	Poor
10	Tiren et al.^10^	36	Poor
11	Lopiz et al.^11^	36	Poor
12	Muramatsu et al.^12^	38	Poor
13	Renger et al.^13^	40	Poor
14	Wang et al.^14^	65	Fair

## 4 Discussion

### 4.1 Anatomy of acromion

The complication may be related to the morphology of the lower aspect of the acromion. Optimizing the shape and length of hook plate can avoid complications.^2^ A total of 102 patients underwent measurement of the lateral acromial angle (α) between the superior surface of the distal clavicle and the inferior facet of the acromion on the coronal plane. Acromion coronal angle (β) was measured between the inferior facet of the acromion and a line from the superior and inferior margin of a glenoid cavity on the coronal plane. Large distal clavicle α > 40° and β < 60° are at the highest risk of impingement.^16^

### 4.2 Design of hook plate

The focus is on the design of the plate, which has a hook portion designed to prevent inferior displacement of the injured arm. Hook retention in the subacromial space results in impingement of the supraspinatus tendon and focal loading of the subacromial surface. Spanning hook prevents the normal scapulothoracic movement of the shoulder girdle, thus increasing the risk of persistent shoulder pain up to 3.6 times. In contrast to locking plate, hook plate can cause skin irritation. Skin irritation is expected in 50% of patients, with one-third of them requiring removal of the implant.^1^

Hook is locked under the acromion. The wider lateral part of the plate provides screw fixation. Rotational movement occurs between the clavicle and scapula during flexion and abduction of the humerus. Hook plate does not restrict this rotational movement.^12^ Wider hook and proper hook plate size can lead to better outcomes. According to a clinical study, forcible fixation of the plate to the clavicle may result in hook migration.^12^

Hook plate has a high mechanical stability due to the hook into the subacromial gap. The plate has combination screw holes for different types of screws. However, this stability comes at the cost of subacromial impingement, bursitis, and rotator cuff injury with a reported incidence of 5%–68%.^17^ The hook stays behind the acromioclavicular joint which acts as a lever and maintains fracture reduction. However, this not only results in limited arm abduction but also affects acromion and induces discomfort.^8^

According to referred biomechanical study, greater depth of hook in acromion will result in less stress on acromion. The clinical application of these findings is that the hook plate is available in different depths i.e., 12, 15, and 18 mm. Depending on the patient's anatomy, if both sizes can be accommodated, the deeper size should be selected to reduce stress on the acromion.^17^ However, the clinical applicability of this study remains debatable. In our opinion, the incidence of impingement is multifactorial, influenced by patient-related factors, implant characteristics, and surgical technique. Regarding patient factors, the acromial angle is a primary consideration, and the depth of hook plate can be optimized using the methods described above. In addition, based on our experience, positioning hook plate more posteriorly helps reduce the risk of impingement.

AO clavicle hook plate is designed in a way that the hook passes below the acromion but remains posterior to the acromioclavicular joint. The hook is gradually widened secondary to rotational movement of the clavicle and Acromioclavicular joint, which sometimes can result in painful shoulder movements.^16^ The advantage of hook plate is that it prevents the rotational movement during flexion and abduction movement of the humerus.^10^ At the time of surgical intervention, the 15 mm hook plate can be tried with the plate resting without resistance on the lateral surface of the clavicle. If resistance occurs, fracture reduction should be reassessed and an 18 mm plate should be tried.^11^

Large lateral acromial angle was defined as distal clavicle acromion coronal angle > 40° and acromion coronal angle <60°. This large angle was attributed to painful post-operative shoulder movements. According to the study by Muramatsu et al.,^12^ the possible solution in this case is to prioritize the removal of the implant after the fracture has healed. This removal of the plate before 6 months contributes to shoulder functional outcomes compared to delayed removal based on evidence in the literature.^12^

### 4.3 Biomechanical analysis

Deep implantation of the clavicle hook plate reduces the stress on the clavicle and the forces applied to the acromion. Three different types of clavicle hook plates were used with the hook plate depth of 12, 15, and 18 mm. There is an increased risk of peri-implant fracture due to increased stress on the clavicle and acromiolysis of the acromion. Therefore, if multiple depths of plates are available, plates with deeper hook plate depths should be used.^17^

### 4.4 Treatment rationale based on expert consensus

An algorithm has been suggested based on expert consensus which divides the clavicle fracture into stable and unstable types. Neer type I and III were classified as stable fractures which are suitable for conservative treatment. In contrast, Neer type IIA, IIB, and V fractures are deemed unstable, warranting operative intervention. Depending on the size of the lateral fragment, patients can be offered locking plate fixation or hook plate for these unstable injuries.^18^

### 4.5 Locking plate design

Based on the evidence in the literature, locking plate has a similar union rate compared to hook plate but it may not require routine removal due to the lack of risk of impingement. Different types of locking plates have been documented in the literature. In this referred study, different types of locking plates have been used, like pre-contoured locking plate, small fragment, dual mini fragment locking plate, and mini fragment locking plate.^1^ In another referred study, the pre-contoured locking plate was used, and multiple screws were used in different directions and optionally up to use as many as 6 small locking screws.^3^^,^^15^

Plate fixation for the clavicle is difficult in general considering the S shape of bone. In the case of a lateral end of clavicle fracture, the size of the lateral fragment possesses an additional surgical challenge. The lateral fragment is often small or of poor quality, making it difficult to achieve stable fixation with a plate and screw system. Additionally, the fixation is subjected to the same forces that cause fracture displacement, and therefore, there is a potential risk of fixation failure in cases of suboptimal fixation.^14^

In a non-randomized control trial comparing the hook and locking plate, one-third of the patients needed implant removal, and 50% suffered from implant-related symptoms.^9^ In another study by Ochen et al.,^8^ the lateral end of the clavicle fracture was immobilized with locking plate with lateral extension, and the implant was removed in 42% (20 patients) of these cases. This can be explained according to the subcutaneous position of the clavicle. It is also worth noting that for locking plate, removal of the implant may be an option depending on the patient's symptoms, but in the case of hook plate, the broader consensus in our experience is to plan for the removal of the implant after the fracture has healed.

### 4.6 Length of the lateral fragment

In our experience, the lateral fragment size is of paramount importance for choosing the types of implants. Small lateral fragments do not provide adequate support for locking/cortical screws, leading to the risk of instability or fixation failure. In our cohort of patients, the mean length of lateral fragment was 9 mm (3–15 mm) which limits the use of locking plate fixation, making hook plate a more appropriate choice. It is worth mentioning that based on our search only 2 studies have documented the length of lateral fragment. However, as mentioned in the study, the choice of the implant was based on the surgeon's choice and was not dependent on the fracture pattern or length of the lateral fragment.^8^

In the case of hook plate fixation, repair of the coracoclavicular ligament is not mandatory as the stability of fixation is supported by a hook along with plate screws thus providing more advantageous stability compared to conventional plating which relies solely on the quality and size of the lateral fragment.^12^ In our limited experience of the use of hook plates, we have not repaired any coracoclavicular ligaments during our fixation.

As per the referred literature, if the clavicular bone block was >20 mm, locking plate can be used. When using locking plate, 4 screws should be inserted in the distal fragment and 3 in the proximal fracture block. If hook plate is used, 3 screws should be implanted in the proximal fracture block; if the distal fragment is stable after reduction, no screws are needed; otherwise, 2 or 3 screws should be implanted.^14^

### 4.7 Complications of hook plate

#### 4.7.1 Hook migration

Hook migration was reported in 84% of cases in the reference study. However, the rehabilitation program did not lead to the migration of the hook. This migration should be addressed by the removal of an implant at 6 months.^16^

#### 4.7.2 Osteolysis

Şükür et al.^2^ suggested a high osteolysis rate of 62%. However, these changes were reversible once the implant was removed.^5^ In another study suggested the mean time of implant removal was 4 months (3–7 months).^7^ However, in our experience, there was no evidence of osteolysis in our patients, probably because we removed the implants before 6 months (5–6 months).

#### 4.7.3 Impingement syndrome

In another study, impingement was documented to be 32% and subacromial osteolysis to be 25%.^11^ In our experience, we are off the view that impingement following hook plate is mechanical and can be confirmed clinically with failure to abduct the arm beyond 90°. The normal function of the shoulder was restored in 1 of our patients following the removal of the plate.

#### 4.7.4 Peri-implant fractures

According to 1 biomechanical study, the use of a longer hook plate with more holes reduces the forces acting on the clavicle, thus decreasing the incidence of acromion osteolysis and peri-implant fractures.^6^ This hypothesis was supported by 1 case in a case series by Şükür et al.^2^ with using 4-hole plates, which led to the peri-implant fracture. Another meta-analysis showed that the use of hook plate increased the risk of complications by 11 times.^5^ Peri-implant fracture with fracture medial to implant is associated with retained implant following fracture healing.^11^

Although the study helps in giving a better understanding of the use and choice of these implants, there are some limitations. However, it is worth mentioning that these are not the only 2 methods being considered for fracture fixation. There are limited case series and evidence reported on the use of locking plates in conjunction with other soft tissue procedures as well as soft tissue procedures alone. However, these studies were not considered in this systematic review.

In conclusion, it is worth mentioning that not only the quality of available literature is poor in terms of more level IV studies. Moreover, the documented risk of complications and outcome measures are also heterogeneous and it is hard to draw a solid or definite conclusion from the available literature. However, surgeons’ preferences and familiarity with the technique should be considered. Patients should be made aware of the risks and benefits of surgery. Pre-operative planning for the size of the lateral fragment should be kept in mind when choosing between the 2 implants. In 2019, a study by Li et al.^19^ supported our conclusions, stating that distal clavicle locking plates for Neer type II distal clavicle fractures resulted in better functional recovery of the shoulder joint and fewer complications related to pain and limited abduction compared with clavicle hook plate. The use of hook plate has been associated with a high union rate but at the cost of implant-related complications. Hook plate and locking plate are 2 of the many treatment options for lateral end of clavicle fracture fixation, and both have comparable healing rates. However, a few complications reported in the literature are also noteworthy.

A multi-center prospective randomized controlled trial should be conducted to determine the ideal fixation method, thereby enhancing the quality of evidence. It is crucial to consider the size of the lateral fragment to guide surgeons in adopting an evidence-based approach for managing fractures of the lateral end of the clavicle. This should be a key consideration in future research.

However, a practical challenge lies in developing and agreeing upon a standardized protocol for selecting the appropriate implant based on fracture patterns, fragment size, and fracture type. Establishing clear inclusion criteria for the 3 study groups is essential to avoid confusion and improve outcomes.

## CRediT authorship contribution statement

**Ravi Patel:** Writing – Review & Editing, Supervision, Project Administration. **Muhammad Murtaza Khan:** Writing – Original Draft, Writing – Review & Editing, Visualization. **William Gibson:** Writing – Review & Editing. **Robin Banerjee:** Writing – Review & Editing. **Asif Pardiwala:** Conceptualization, Supervision, Writing – Review & Editing.

## Ethical statement

There are no ethical conflicts associated with this study.

## Funding

There is no funding associated with this study.

## Declaration of competing interest

The author and co-author have no conflict of interest and they have nothing to declare. There is no funding associated with this study.
